# Treating hypertension with single pill combinations: a simple strategy to save costs for the patients and payers

**DOI:** 10.1097/HJH.0000000000004050

**Published:** 2025-06-16

**Authors:** Miriam Pikkemaat, Emily R. Atkins, Anthony Rodgers, Aletta E. Schutte

**Affiliations:** a Center for Primary Healthcare Research, Department of Clinical Sciences, Malmö, Lund University, Malmö; b University Clinic Primary Care Skåne, Region Skåne, Sweden; c The George Institute for Global Health; d School of Population Health, University of New South Wales, Sydney, NSW, Australia

**Keywords:** blood pressure, cost comparison, cost minimization analysis, drug combinations, fixed-dose combination, single pill combination

## Abstract

**Objectives:**

Our aim was to compare direct costs for single pill combinations (SPCs) and free-drug combinations for hypertension treatment.

**Methods:**

We focused on Australia as a case study and reviewed total costs, and for the patient and government. We reviewed the Australian “Pharmaceutical Benefits Scheme item drug map” considering different thresholds for the government safety net. Total costs included medicine costs and pharmacy fees.

**Results:**

For patients, SPCs always cost less than free-drug combinations, with greatest savings for general patients before reaching safety net (averaging 30%). For government, SPCs cost on average less than free-drug combinations, for Concession Card holders both before (averaging 11%) and after reaching safety net (averaging 26%) and in general patients after safety net (averaging 11%). There was a slight increase in costs (16%) for the government for patients before reaching safety net. All findings were driven by savings in dispensing fees, the main cost of supply, also after the recent introduction of 60-day dispensing.

**Conclusion:**

Single pill combinations, instead of free-drug combinations, result in cost saving for both patient and government in almost all cases and often these savings are large. SPC cost savings should be factored into prescribing decisions, both for people receiving multiple pills and people starting treatment.

## INTRODUCTION

Raised blood pressure is an increasing concern and causes over 10 million deaths each year globally [1]. The cornerstone of blood pressure control is the adequate prescription and adherence to blood pressure lowering therapy. All recent major international guidelines [2–5] recommend dual single pill combinations (SPCs) as first-line therapy for hypertension, both to initiate treatment and for patients taking multiple blood pressure medications. Treatment inertia appears when the health-care provider does not initiate or intensify therapy appropriately when therapeutic goals are not reached [6]. SPC therapy has demonstrated greater effectiveness in blood pressure lowering, addressing treatment inertia and improved medication adherence [7]. A recently published study predicted that the number of saved lives over the next 30 years across 24 low- and middle-income countries by using SPCs instead of monotherapy in hypertension was an impressive 564 000, which is significantly greater than the 430 000 lives predicted using free-drug combinations in dual therapy [8].

In this article, we will focus on Australia as a case study. One third of Australian adults have hypertension and 68% of them remain uncontrolled [9]. Looking at control rates, Australia compares poorly with similar high-income countries, such as Canada [10]. We therefore published a call-to-action in 2022 [10], followed by the establishment of the National Hypertension Taskforce of Australia aiming to significantly improve blood pressure control [11]. The last Australian Hypertension Guideline was released in 2016 [12] and is outdated in key areas. It recommends starting hypertension treatment with low-moderate monotherapy, with consideration of dual therapy and is now finally about to be updated [13].

Still, in Australia, the current pharmaceutical benefits scheme (PBS) restrictions do not allow physicians to initiate subsidized antihypertensive therapy with a SPC as recommended internationally, and the current drug labels of all but one antihypertensive SPC state that “the treatment must not be for the initiation of antihypertensive therapy”. Using SPCs as a second step in hypertension treatment is allowed, but despite the availability of a lot of different SPCs, there is an underuse of them in hypertension treatment in Australia [14]. A recent study [15] showed that in 2022 $1.2 billion was spent on the management of hypertension in Australia, with 51% of the costs being pharmacy-related fees. Forty-one percentage of the costs were from patient out-of-pocket payments suggesting that out-of-pocket costs likely remain a key barrier to blood pressure control due to impacting medication adherence. Hence, reducing out-of-pocket medication costs can lead to improvements in medication adherence [16].

Multiple meta-analyses have previously shown that SPCs improve medication adherence [7,17,18] and medication persistence [7,17,18]. Additionally, a recent large outcomes claims data analysis has demonstrated that SPCs reduce cardiovascular events and all-cause mortality in patients with hypertension [19]. These should be convincing arguments for the extended use of SPCs. A barrier for both changing prescriptions and an argument against changing drug labels and PBS prescriptions might be the perception that SPCs are more expensive than their equivalent free-drug combinations. When new drugs enter the market, they often come with high costs, as brand-names. But since the introduction of SPCs about 15 years ago, their patents have expired [15]. Generics are now available for all but one SPC in Australia and prices have decreased substantially.

This shows the need for a study with specific focus on direct cost savings, for both the patients and the government.

A previous Australian study found SPCs to be cost-effective, primarily benefiting patients [11]. Recent reductions in out-of-pocket costs [16] and extended dispensing durations from 30 to 60 days in Australia are expected to impact these costs, though no studies have assessed this yet. Cost savings extend beyond medication expenses to improved health outcomes, adherence, and reduced healthcare use. A key concern for greater SPC uptake and subsidized antihypertensive initiation is affordability for both patients and the government, influencing prescribing decisions and medication adherence. Few studies compare SPC prices to free-drug combinations [8], mostly in low-income countries.

We therefore aimed in this cost minimization analysis, to specifically study direct cost savings by comparing current costs for SPCs for the treatment of hypertension and free-drug combinations, in total, for the patient and the government, focusing on Australia.

## METHODS

We conducted a cost-minimization analysis of single pill combination blood pressure lowering in comparison to the equivalent free-drug combination. As both contain equivalent active ingredients and doses, the efficacy of both is considered equivalent. Costs are 2024 Australian dollars. The setting is the Australian Health System. The perspective adopted is the health system payer perspective to consider government costs and patient copayments. The outcomes of this analysis were the total costs, the total cost per mmHg, the cost to patient (by beneficiary type), and the cost to government (by beneficiary type). No individual patient data was collected. For a detailed example in Table 1, we analyzed costs for amlodipine (5 mg), valsartan (160 mg), and hydrochlorothiazide (25 mg) in their standard doses as they are widely used, have well proven efficacy and are available as single pills in Australia for both dual and triple combinations.

**TABLE 1:** Cost comparison – for patients and government, between single pill combinations and free drug combinations, per pill price (in Australian dollar, AUD) multiplied by 60 to facilitate comparison

Medicines	free-drug combination or single pill combination (SPC)	What does it cost?			Who pays?							
		Cost for 60-day supply (AUD)			Paid directly by patients (AUD)				Paid by federal government (AUD)			
		Medicine^a^	Pharmacy fees^b^	Total cost^c^	General		Concession Card^d^		General		Concession Card^d^	
					Before safety net^e^	After safety net^e^	Before safety net^e^	After safety net^e^	Before safety net^e^	After safety net^e^	Before safety net^e^	After safety net^e^
Valsartan 160 mg	One drug	17.21	13.92	31.13	31.13	8.25	8.25	0	0	22.88	22.88	31.13
Amlodipine 5 mg	One drug	5.42	12.99	18.41	18.41	7.70	7.70	0	0	10.71	10.71	18.41
Hydrochlorothiazide 25 mg^f^	One drug	4.89	3.90	8.79	8.79	2.31	2.31	0	0	6.48	6.48	8.79
Valsartan 160 mg + Amlodipine 5 mg	Free-drug combination	22.63	26.91	49.54	49.54	15.95	15.95	0	0	33.59	33.59	49.54
	SPC	21.46	13.92	35.37	31.60	8.25	8.25	0	3.77	27.12	27.12	35.37
Valsartan 160 mg + Amlodipine 5 mg + Hydrochlorothiazide 25 mg	Free-drug combination	27.51	30.82	58.33	58.33	18.26	18.26	0	0	40.07	40.07	58.33
	SPC	27.95	13.92	41.86	31.60	8.25	8.25	0	10.26	33.61	33.61	41.86

a Costs for medicine = ex-manufacturer price + wholesale markup.

b Pharmacy fees = administration, handling and infrastructure fee + dispensing fee calculated for 60 days.

c Total cost = Dispensed price maximum quantity. Each product's per pill price has been multiplied by 60 to facilitate comparison.

d Concession card – given to patients holding one of the following cards: pensioner concession card, commonwealth seniors health card, healthcare card, Department of Veterans’ Affairs White, Gold, or Orange Card.

e Safety net – General patient safety net threshold is $1647.90, Concessional Safety Net threshold is $277.20 (since 2024).

f Costs for Hydrochlorothiazide calculated based on a 200 pack 25 mg.

### Medicine identification and script volumes

The medicines were identified using the ‘Body Systems’ ATC codes in the PBS schedule. The codes we used were C09A ‘ACE Inhibitors, plain’, C09B ‘ACE Inhibitors, combinations’, C09C ‘Angiotensin II receptor blockers (ARBs), plain’, and C09D ‘Angiotensin II receptor blockers (ARBs), combinations’. PBS ATC codes are very similar to the WHO ATC but not always identical (https://www.ncbi.nlm.nih.gov/pmc/articles/PMC7647230/). Script volumes were calculated using the item numbers for each medication from the PBS webpage [20] and from the Medicare statistics Pharmaceutical Benefits Schedule Item Reports [21].

### Medicine prices

Prices were collected in March 2024 from the PBS official website [20]. The total costs included the costs for the medicine itself, consisting of the ex-manufacturer price and the Wholesale Markup and the pharmacy fees, consisting of the Administration, Handling and Infrastructure fee and the Dispensing fee. To ensure fair comparison, we calculated the per-pill cost for all available pack sizes and selected the lowest-cost option, multiplying it by 60. Pack sizes vary (e.g., 28, 30, or 100 pills), with maximum supply limits allowing two packs (56, 60, or 200 pills).

### Patient out-of-pocket costs

In Australia, patients pay up to 31.60 AUD per prescription (7.70 AUD for Concession Card holders, including pensioners and low-income earners). General patients reach the safety net at 1647.90 AUD, reducing costs to 7.70 AUD per prescription. Concession holders reach 277.20 AUD, after which prescriptions are free for the rest of the year [22].

### Total cost per mmHg systolic blood pressure reduction from 150 mmHg

As an additional analysis, we calculated the total cost per mmHg systolic blood pressure reduction to highlight the broader benefits of single-pill combinations. Based on a previous meta-analysis [23], we used mean reductions of 9 mmHg for one drug, 16 mmHg for two drugs, and 23 mmHg for combination therapy at standard doses. These values, derived from patients with an initial systolic blood pressure of 150 mmHg, align with Table 3 in the referenced study.

### Ethics approval and reporting guideline

No patient data was collected, and no patients were contacted. Ethics approval was not required, a waiver was not sought. The study is reported in accordance with the reporting guidelines of the Consolidated Health Economic Evaluation Reporting Standards [24] (CHEERS; checklist is provided in the Appendix).

## RESULTS

**FIGURE 1:**
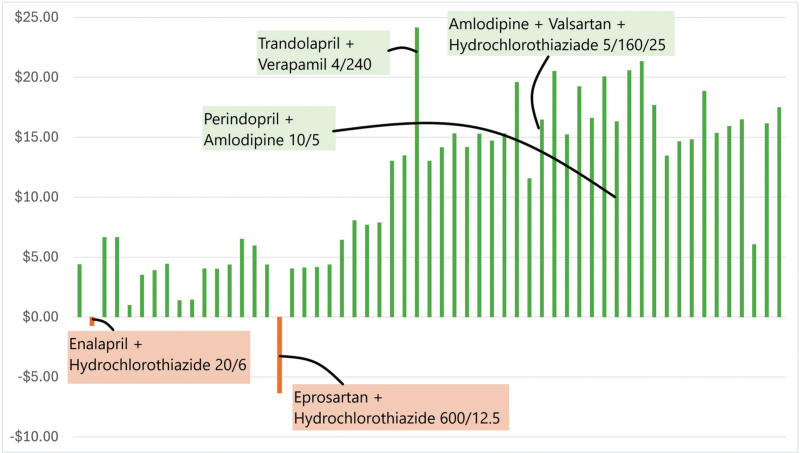
Absolute price reduction in AUD using single pill combinations (SPCs) instead of free-drug combinations, for the 57 different available SPCs.

**FIGURE 2:**
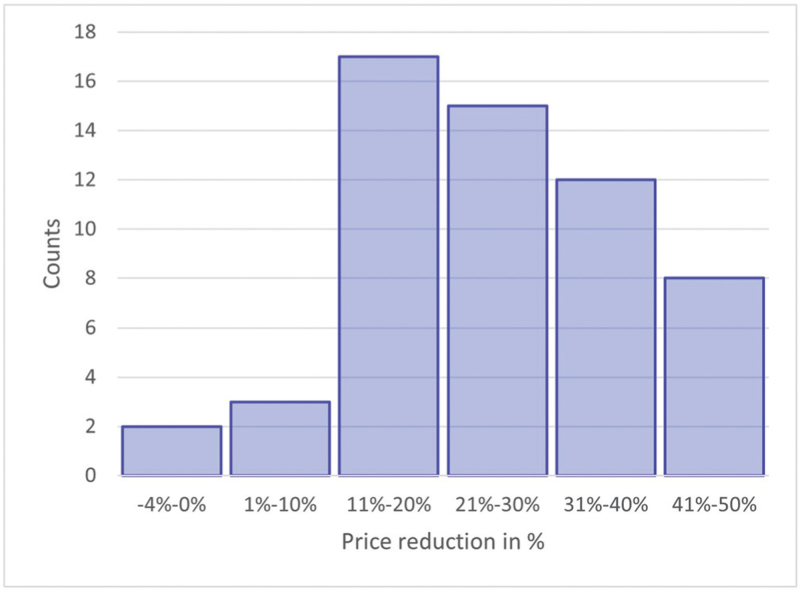
Price reduction in % for supply of 60 days of medicines using single pill combinations (SPCs) instead of the free-drug combinations, for 57 available SPCs.

A total of 19 dual (with 47 dose options) and 2 triple SPCs (with 10 dose options) were listed on the PBS. This resulted in 57 different estimates of the cost of providing medicine as SPCs compared to the free-drug combinations. The cost calculations for an example SPC are shown in Table 1, the mean cost savings in Table 2 and a summary of all price reductions are shown in Figs. 1 and 2. The complete cost calculations are shown in the supplementary material (Table S1, Supplemental Digital Content).

**TABLE 2:** Mean cost savings for patients and government, according to eligibility status of subsidy, when using single pill combinations instead of free-drug combinations, for the 57 available single pill combinations

	Patients		Government	
	Absolute Saving (AUD)	Percentage Saving	Absolute Saving (AUD)	Percentage Saving
General before safety net	12.91	30%	-2.03	-16%
General after safety net	7.79	48%	3.09	11%
Concession before safety net	7.79	48%	3.09	11%
Concession after safety net	0	0%	10.88	26%

**TABLE 3:** Cost comparison per mmHg systolic blood pressure reduction between single pill combinations and free drug combinations in Australian Dollar (AUD)

		Total cost per mmHg^1^
Valsartan 160mg	one drug	3.46 (31.13/9)
Amlodipine 5mg	one drug	2.05 (18.41/9)
Hydrochlorothiazide 25mg^7^	one drug	0.98 (8.79/9)
Valsartan 160 mg + Amlodipine 5mg	free-drug combination	3.10 (49.54/16)
	SPC	2.21 (35.37/16)
Valsartan 160 mg + Amlodipine 5 mg + Hydrochlorothiazide 25mg	free-drug combination	2.54 (58.33/23)
	SPC	1.82 (41.86/23)

^1^Calculated per mmHg reduction. For one drug: the cost per 9mmHg systolic blood pressure reduction, for two drugs the cost per 16 mmHg reduction, and for three drugs the cost per 23 mmHg reduction [17].

### Script volumes

The blood pressure lowering drugs with the highest prescription volume in Australia in 2022–2023 were perindopril (Top 5 of all PBS medicines with a total prescription volume of 7,092,072) and amlodipine (Top 10, total prescription volume) [24]. The most dispensed SPC with the highest prescription volume was perindopril + amlodipine with a total of 3 183 857 [25].

We extracted the prescribed volumes from March 2023 to February 2024 for amlodipine, hydrochlorothiazide and valsartan, being available both as dual and triple combinations. The single drug prescription volumes were 3 209 454 (amlodipine), 367 825 (valsartan) and 2244 (hydrochlorothiazide), totalling 3 579 523 prescriptions during the 12 months. The different dual SPCs (amlodipine + valsartan or valsartan + hydrochlorothiazide) correspond to a total of 655 275 and the triple combinations to a total of 418 027 prescriptions.

### Total cost reductions

Analyzing the total costs for all 57 available combinations shows that using SPCs instead of free-drug combinations leads to a mean cost reduction of 10.88 Australian dollar (AUD) for each 60-day period corresponding to a percentage reduction of 25.6% (Table S1, Supplemental Digital Content).

The maximum absolute cost reduction was 24.17 AUD (Fig. 1, Figure S1, Supplemental Digital Content) and the maximum percentage reduction 47.6% (Fig. 2, Table S1, Supplemental Digital Content). Only in 2 of the 57 medicines using SPCs would lead to an increase in total costs (6.33 AUD for Eprosartan + hydrochlorothiazide (no generic available), and 0.70 AUD for Enalapril + hydrochlorothiazide) (Fig. 1). Using perindopril and amlodipine (the most common combination) as SPC instead of free-drug combination gives a saving of 38–46% (15–18 AUD), depending on the doses (Table S1, Supplemental Digital Content).

### Costs for patients and for the government, according to eligibility status for subsidy

The cost calculations according to subsidy eligibility for an example single pill combination are shown in Table 1, the cost calculations for all 57 available combinations are shown in the supplement (Table S1, Supplemental Digital Content).

The mean costs for SPCs are always lower than for free-drug combinations for all patients, independent of subsidy eligibility. The savings are greatest for General patients who have not reached the safety net (a mean of 13 AUD lower out-of-pocket costs per script, which is a saving of 30%) (Table 2). Savings are also seen clearly for both General patients after the safety net, and for Concession card holders before reaching the safety net (in both a mean of 48% lower costs).

For the government, SPC prescriptions result in cost savings when funding treatments for General Patients after the safety net and Concession Card holders before the safety net (for both a mean of 11% lower costs) and greater savings for Concession Card holders after the safety net (mean absolute saving of 11 AUD, a 26% saving). For the government there is a cost increase for General patients before the safety net (absolute mean increase of 2 AUD; 16%) (Table 2). This cost increase is accompanied by substantial savings for the patient and is mainly driven by pharmacy fees that exceed the cost of medicines themselves in most cases. In the given example in Table 1, for the government there is a cost increase of AUD 3.77 for the dual and AUD 10.26 for the triple, which are accompanied by a substantial cost saving for out-of-pocket payment by the patient (AUD 17.94 and AUD 26.73).

Sixty-day dispensing has not been implemented for all patients, and in situations where 30-day dispensing remains, the proportional cost savings would be even greater for SPCs compared to free-drug combinations. For example, valsartan 160 + amlodipine 5 saves a general patient 35.53% as a single pill combination with 30-day dispensing, compared to 28.6% with 60-day dispensing.

### Costs per mmHg blood pressure reduction

An example for the costs per mmHg systolic blood pressure reduction comparing SPCs and free drug-combinations is shown in Table 3.

A comparison of the costs per mmHg systolic blood pressure reduction for the different SPCs and free drug-combinations are shown in Figure S2, Supplemental Digital Content and Table S1, Supplemental Digital Content. In 96.5% (55/57) of the combinations the costs per mmHg blood pressure reduction are lower when using SPCs. The mean cost per 1 mmHg systolic blood pressure lowering was 2.37 AUD for free-drug combinations and 1.75 AUD for SPCs, which is 26.2% lower when using SPCs.

## DISCUSSION

Our cost minimization analyses demonstrate that using SPCs instead of free-drug combinations for the treatment of hypertension leads to a significant cost saving from a health system payer perspective in Australia. Looking at total costs, 55 of the 57 available SPCs had lower costs than their equivalent in free-drug combinations. For one of the other two (eprosartan + hydrochlorothiazide, 6.33 AUD cost increase), no generic was available. The small cost increase (0.70 AUD) for enalapril + hydrochlorothiazide may be reflecting lower competition on that combination product as there are only two brands available on enalapril + hydrochlorothiazide. For the patients, there was a mean saving, independent of subsidy eligibility. For the government there were mean savings for both concession card holders and general patients after reaching the safety net. Only in general patients, before reaching the safety net, there was a slight cost increase for the government when using SPCs, but that was accompanied by major savings for the patient.

Our study was based on Australia as a case study. With diversity among different countries regarding health systems, costs such as pharmacy fees, direct medicine fees, government and infrastructure fees it is challenging to perform direct comparisons between countries. However, our findings from Australia serve as a prompt to evaluate costs and relevant barriers towards implementing SPCs in different contexts. Based on our analysis more international country-specific research is required, as major cost savings are clearly possible.

In Australia, relatively few patients with hypertension are treated with SPCs. In a study from 2018 only one-third of all people taking blood pressure lowering medicines used SPCs [26]. International guidelines clearly recommend the use of SPCs for the initial treatment of hypertension [2–5] but in Australia the drug labels of SPCs typically state that “treatment should not be initiated with this fixed-dose combination” and the PBS restrictions do not allow physicians to initiate subsidized antihypertensive therapy with a SPC, with one exception.

A key reason for the recommendation to use SPCs as initial treatment, is due to benefit that it helps overcome treatment inertia [7]. In general practice, treatment inertia remains one of the most important problems affecting blood pressure control [27]. A recent study evaluating 800 million general practice visits of patients with uncontrolled hypertension showed that only 10.3% of the visits resulted in treatment intensification [28]. In the USA, a major program with a simplified SPC -based protocol led to blood pressure control rates improving from 44% to 80% [29], clearly demonstrating that SPCs should be part of a modern simplified strategy [30].

In Australia, blood pressure control rates of 32% (<140/90 mmHg) compare poorly to other international settings, and these control rates have stagnated for a decade [9,10]. In response to a call-to-action the National Hypertension Taskforce of Australia was launched, which developed a roadmap to improve control rates from 32% to 70% by 2030 [31]. A key focus of the roadmap includes implementation of SPCs. An Australian study using a large national database of electronic medical records from general practice showed a 55% blood pressure control rate for patients diagnosed with hypertension (<140/90 mmHg) [32]. But a third of patients diagnosed with hypertension more than 5 years ago, had no record of being prescribed any antihypertensive medication [32].

Current practice in Australia is to start treatment with monotherapy, with intention to increase dose at follow-up visits instead of adding another drug class [14]. Unfortunately, there is significant treatment inertia by not up-titrating doses adequately, nor adding another drug class, contributing to poor treatment and control in Australia.

To implement SPCs as initial treatment, drug labels in Australia need to be updated. A barrier for changing labels may be the perception that SPCs are more expensive. But since the introduction of SPCs about 15 years ago, their patents have expired [33]. Since generics are now available for all but one SPC, prices have decreased substantially.

However, despite these price reductions, low-income earners may still find out-of-pocket costs to be an important barrier to medicine adherence (in Australia 7.70 AUD per refill for Concession card holders before safety net). When directly comparing the costs between SPCs and free-drug combinations we clearly showed that using SPCs does not lead to increased medicine costs. There are substantial out-of-pocket savings for both General and Concession patients when switching SPCs. For the government switching to SPCs would mean significant savings on Concession patient co-payments and would generate a small cost increase only for General patients before reaching safety net. Costs should therefore not be a barrier for implementing SPCs as a first line treatment for hypertension in Australia. Our analysis clearly shows much better value for money in terms of cost per mmHg systolic blood pressure reduction with SPCs.

One existing key challenge is that drug labels are not updated when products become generic. Australian drug labels for dual SPCs have not been updated in line with evidence to remove restriction of initial use, which flows into reimbursement restrictions. Updating labels and removing reimbursement restrictions to initiate hypertension therapy with SPCs are essential steps to allow general practitioners to follow evidence-based best practice.

Few studies have been published comparing direct costs of SPCs and free-drug combinations and most of them are limited to low-income countries [8]. An Indian study showed that the lowest prices of SPCs and of the sum of components were nearly identical [34]. A recent published study on cost and cost-effectiveness of SPCs showed that the ratio of purchasing prices of SPCs to equivalents as free-drug combinations was low for the minimum prices, meaning that the SPCs were a lot cheaper for Brazil, Bangladesh, Philippines, South Africa, Argentina and Nigeria. The only compared country with higher prices for SPCs in this study was Thailand [8].

Only very few price comparisons seem to have been published for high-income countries. An American study showed that SPC dual therapy was cost-effective versus equivalent dose dual therapy as free-drug combination (ICER $57 000/QALY gained) [35]. In an Italian study, evaluating the lifetime cost-effectiveness profile of a three-drug SPC vs. the corresponding two-pill administration (a two-drug SPC plus a third drug separately) from the Italian payer perspective, the cost reduction associated with the SPC was primarily driven by savings in hospitalization costs, the drug costs themselves were higher for the SPC [36]. Our detailed cost comparisons, based on Australian data, showed large direct cost savings. This in an important argument in strengthening and implementing local guidelines to improve the use of SPCs in hypertension treatment. Further detailed comparisons for other countries could be of interest and we welcome and encourage more studies on this topic.

In Australia, the 2016 hypertension guidelines are about to be updated in accordance with international guidelines [13] and we recommend updating the drug labels of SPCs in Australia to remove reimbursement restrictions to initiate hypertension therapy with SPCs. Increasing the use of SPCs as first-line therapy for blood pressure lowering is one important step to reach the overall aim to improve blood pressure control, both in Australia [31] and internationally.

### Limitations

The analysis presented here is based on the published costs of individual components and SPCs on 27 March 2024. These costs fluctuate over time based on market pressures, policy changes, and the Community Pharmacy Agreement. Some pharmacies provide a $1 discount to the co-payment for consumers under the PBS, but as this is not uniformly available and applied, we have not accounted for it in our estimates. However, the results will hold true while the total cost of a SPC remains lower than the individual components, and while the SPC cost is above the General Patient copayment threshold. If the cost of the SPC drops below the General Patient copayment, then it becomes cost saving to both the patient and the government in comparison to free-drug combinations. The costs for drugs and fees, reimbursement systems but also availability of SPCs vary between different countries. The results of this study are not necessarily applicable to other countries and their healthcare systems which is a limitation of the study. On the other hand, using Australia as a case study can be helpful to other countries to evaluate public health policy related to the implementation and costs of SPCs.

## CONCLUSION

Our findings demonstrate that using SPCs instead of free-drug combinations lead to substantial savings in almost all cases in a detailed analysis in Australia. In addition to the clinical considerations, the large direct cost savings from SPCs should be factored into prescribing decisions, both for people receiving multiple separate pills and people starting treatment.

## ACKNOWLEDGEMENTS

Funding: M.P. is supported by Swedish Governmental Funding of Clinical Research (ALF) and by the Swedish Society of Medicine). A.E.S. is supported by an NHMRC Investigator Grant (APP2017504).

### Conflicts of interest

George Health Enterprises Pty Ltd, the social enterprise arm of The George Institute for Global Health (TGI), has received investment to develop fixed-dose combination products containing aspirin, statin and blood-pressure lowering drugs. TGI holds and have filed applications for method of treatment and composition patents in relation to low and ultra-low-dose fixed-dose combination products for the treatment of hypertension and diabetes, and Professor Rodgers is listed as one of the inventors (Granted: US 10 369 15; US 10 799 487; US 10 322 117; US 11 033 544; US 11 478 462; Pending: US 17/932 982; US 18/446 268; US 17/598 122; US 17/317 614). Professor Rodgers is seconded part-time to George Medicines Pty Ltd (GM), a subsidiary of George Health Enterprises. All staff employed by TGI have an institutional interest to declare with respect to George Health Enterprises. None of the TGI staff have a direct financial interest in these investments. AES received speaker honoraria and/or travel fees from Servier, Novartis, Medtronic, Omron, Sanofi, AstraZeneca, Abbott, and Aktiia.

## Supplementary Material

Supplemental Digital Content
