# Supplementary material for: Treating hypertension with single pill combinations: a simple strategy to save costs for the patients and payers
Source: J Hypertens. 2025 Jun 16;43(9):1478–84. doi: 10.1097/HJH.0000000000004050 (PMC12337932; doi:10.1097/HJH.0000000000004050)
Supplement: Supplemental Digital Content [file jhype-43-1478-s001.docx]

**Supplementary**

**Treating hypertension with single pill combinations: a simple strategy to save costs for the patients and payers**

This supplementary file contains 2 tables, 2 figures and a checklist as a combined word document:

- Table S1a. Prices for free-drug combinations, single pill combinations, Costs per mmHg and price reduction when single pill combinations are used, for all 57 available single pill combinations
- Table S1b. Absolute Cost Saving in AUD and percentage cost savings when single pill combinations are used instead of free-drug combinations, for patients and government, according to eligibility status of subsidy, for all 57 available single pill combinations
- Figure S1. Price reduction in AUD for supply of 60 days of medicines using single pill combinations instead of free-drug combinations, for 57 available single pill combinationsFigure S2. Price reduction per mmHg blood pressure lowering when using single pill combinations instead of free-drug combinations for the 57 different combinations
- Figure S2. Price reduction per mmHg blood pressure lowering when using single pill combinations instead of free-drug combinations for the 57 different combinations
- Cheers 2022 Checklist

| **Table S1a. Prices for free-drug combinations, single pill combinations, Costs per mmHg and price reduction when single pill combinations are used, for all 57 available single pill combinations** | | | | | | | | | | | |
| --- | --- | --- | --- | --- | --- | --- | --- | --- | --- | --- | --- |
|  | **Prices for free-drug combinations (in AUD)** | | | |  | **Cost per mmHg** | | **Price reduction when single pill combination used** | | | |
| **Single Pill Combinations** | **Medi-cation 1** | **Medi-cation 2** | **Medi-cation 3** | **Sum**  **Medication 1-3** | **Price single pill combination (AUD)** | **Free-drug combination** | **Single pill combination** | **Absolute price reduction (AUD)** | **% Price reduction** | **Absolute price reduction per mmHg** | **% Price reduction per mmHg** |
| PERINDOPRIL + INDAPAMIDE 2.5/0.625 | 18.41 | 4.40 |  | 22.81 | 18.41 | 1.43 | 1.15 | 4.40 | 19.29% | 0.28 | 19.3% |
| ENALAPRIL + HYDRO-CHLOROTHIAZIDE 20/6 | 20.83 | 2.20 |  | 23.03 | 23.73 | 1.44 | 1.48 | -0.70 | -3.04% | -0.04 | -3.0% |
| PERINDOPRIL + INDAPAMIDE 5/1.25 | 19.79 | 8.80 |  | 28.59 | 21.91 | 1.79 | 1.37 | 6.68 | 23.36% | 0.42 | 23.4% |
| PERINDOPRIL + INDAPAMIDE 4/1.25 | 19.79 | 8.80 |  | 28.59 | 21.91 | 1.79 | 1.37 | 6.68 | 23.36% | 0.42 | 23.4% |
| FOSINOPRIL + HYDRO-CHLOROTHIAZIDE 20/12.5 | 39.40 | 4.39 |  | 43.79 | 42.78 | 2.74 | 2.67 | 1.01 | 2.31% | 0.06 | 2.3% |
| VALSARTAN + HYDRO-CHLOROTHIAZIDE 80/12.5 | 27.33 | 4.39 |  | 31.72 | 28.21 | 1.98 | 1.76 | 3.51 | 11.07% | 0.22 | 11.1% |
| VALSARTAN + HYDRO-CHLOROTHIAZIDE 160/12.5 | 31.13 | 4.39 |  | 35.52 | 31.60 | 2.22 | 1.98 | 3.92 | 11.04% | 0.25 | 11.0% |
| VALSARTAN + HYDRO-CHLOROTHIAZIDE 320/12.5 | 35.99 | 4.39 |  | 40.38 | 35.93 | 2.52 | 2.25 | 4.45 | 11.02% | 0.28 | 11.0% |
| QUINAPRIL + HYDRO-CHLOROTHIAZIDE 20/12.5 | 35.96 | 4.39 |  | 40.35 | 38.96 | 2.52 | 2.44 | 1.39 | 3.44% | 0.09 | 3.4% |
| QUINAPRIL + HYDRO-CHLOROTHIAZIDE 10/12.5 | 34.22 | 4.39 |  | 38.61 | 37.14 | 2.41 | 2.32 | 1.47 | 3.81% | 0.09 | 3.8% |
| OLMESARTAN + HYDRO-CHLOROTHIAZIDE 40/12.5 | 27.93 | 4.39 |  | 32.32 | 28.27 | 2.02 | 1.77 | 4.05 | 12.53% | 0.25 | 12.5% |
| OLMESARTAN + HYDRO-  CHLOROTHIAZIDE 20/12.5 | 21.23 | 4.39 |  | 25.62 | 21.59 | 1.60 | 1.35 | 4.03 | 15.73% | 0.25 | 15.7% |
| TELMISARTAN + HYDRO-CHLOROTHIAZIDE 80/12.5 | 23.13 | 4.39 |  | 27.52 | 23.13 | 1.72 | 1.45 | 4.39 | 15.95% | 0.27 | 16.0% |
| VALSARTAN + HYDRO-CHLOROTHIAZIDE 320/25 | 35.99 | 8.79 |  | 44.78 | 38.26 | 2.80 | 2.39 | 6.52 | 14.56% | 0.41 | 14.6% |
| VALSARTAN + HYDRO-CHLOROTHIAZIDE 160/25 | 31.13 | 8.79 |  | 39.92 | 33.95 | 2.50 | 2.12 | 5.97 | 14.95% | 0.37 | 15.0% |
| CANDESARTAN + HYDRO-CHLOROTHIAZIDE 32/12.5 | 21.59 | 4.39 |  | 25.98 | 21.59 | 1.62 | 1.35 | 4.39 | 16.90% | 0.27 | 16.9% |
| EPROSARTAN + HYDRO-CHLOROTHIAZIDE 600/12.5 | 44.52 | 4.39 |  | 48.91 | 55.24 | 3.06 | 3.45 | -6.33 | -12.94% | -0.40 | -12.9% |
| CANDESARTAN + HYDRO-CHLOROTHIAZIDE 16/12.5 | 21.25 | 4.39 |  | 25.64 | 21.59 | 1.60 | 1.35 | 4.05 | 15.80% | 0.25 | 15.8% |
| TELMISARTAN + HYDRO-CHLOROTHIAZIDE 40/12.5 | 19.73 | 4.39 |  | 24.12 | 19.98 | 1.51 | 1.25 | 4.14 | 17.16% | 0.26 | 17.2% |
| IRBESARTAN + HYDRO-CHLOROTHIAZIDE 300/12.5 | 20.51 | 4.39 |  | 24.90 | 20.73 | 1.56 | 1.30 | 4.17 | 16.75% | 0.26 | 16.7% |
| IRBESARTAN + HYDRO-CHLOROTHIAZIDE 150/12.5 | 18.41 | 4.39 |  | 22.80 | 18.41 | 1.43 | 1.15 | 4.39 | 19.25% | 0.27 | 19.3% |
| OLMESARTAN + HYDRO-CHLOROTHIAZIDE 40/25 | 27.93 | 8.79 |  | 36.72 | 30.27 | 2.30 | 1.89 | 6.45 | 17.57% | 0.40 | 17.6% |
| TELMISARTAN + HYDRO-CHLOROTHIAZIDE 80/25 | 23.13 | 8.79 |  | 31.92 | 23.84 | 2.00 | 1.49 | 8.08 | 25.31% | 0.51 | 25.3% |
| CANDESARTAN + HYDRO-CHLOROTHIAZIDE 32/25 | 21.59 | 8.79 |  | 30.38 | 22.67 | 1.90 | 1.42 | 7.71 | 25.38% | 0.48 | 25.4% |
| IRBESARTAN + HYDRO-CHLOROTHIAZIDE 300/25 | 20.51 | 8.79 |  | 29.30 | 21.41 | 1.83 | 1.34 | 7.89 | 26.93% | 0.49 | 26.9% |
| AMLODIPINE + VALSARTAN 10/320 | 18.41 | 35.99 |  | 54.40 | 41.37 | 3.40 | 2.59 | 13.03 | 23.95% | 0.81 | 24.0% |
| AMLODIPINE + VALSARTAN 5/320 | 18.41 | 35.99 |  | 54.40 | 40.92 | 3.40 | 2.56 | 13.48 | 24.78% | 0.84 | 24.8% |
| TRANDOLAPRIL + VERAPAMIL 4/240 | 33.18 | 40.24 |  | 73.42 | 49.25 | 4.59 | 3.08 | 24.17 | 32.92% | 1.51 | 32.9% |
| AMLODIPINE + VALSARTAN 10/160 | 18.41 | 31.13 |  | 49.54 | 36.50 | 3.10 | 2.28 | 13.04 | 26.32% | 0.82 | 26.3% |
| AMLODIPINE + VALSARTAN 5/160 | 18.41 | 31.13 |  | 49.54 | 35.37 | 3.10 | 2.21 | 14.17 | 28.60% | 0.89 | 28.6% |
| AMLODIPINE + VALSARTAN + HYDROCHLOROTHIAZIDE 10/320/25 | 18.41 | 35.99 | 8.79 | 63.19 | 47.86 | 2.75 | 2.08 | 15.33 | 24.26% | 0.67 | 24.3% |
| AMLODIPINE + VALSARTAN + HYDROCHLOROTHIAZIDE 10/160/12.5 | 18.41 | 31.13 | 4.39 | 53.93 | 39.74 | 2.34 | 1.73 | 14.19 | 26.31% | 0.62 | 26.3% |
| AMLODIPINE + VALSARTAN + HYDROCHLOROTHIAZIDE 5/160/12.5 | 18.41 | 31.13 | 4.39 | 53.93 | 38.63 | 2.34 | 1.68 | 15.30 | 28.37% | 0.67 | 28.4% |
| AMLODIPINE + VALSARTAN 5/80 | 18.41 | 27.33 |  | 45.74 | 31.02 | 2.86 | 1.94 | 14.72 | 32.18% | 0.92 | 32.2% |
| AMLODIPINE + VALSARTAN + HYDROCHLOROTHIAZIDE 10/160/25 | 18.41 | 31.13 | 8.79 | 58.33 | 43.00 | 2.54 | 1.87 | 15.33 | 26.28% | 0.67 | 26.3% |
| OLMESARTAN + AMLODIPINE 40/10 | 27.93 | 18.41 |  | 46.34 | 26.73 | 2.90 | 1.67 | 19.61 | 42.32% | 1.23 | 42.3% |
| TRANDOLAPRIL + VERAPAMIL 2/180 | 24.16 | 23.67 |  | 47.83 | 36.25 | 2.99 | 2.27 | 11.58 | 24.21% | 0.72 | 24.2% |
| AMLODIPINE + VALSARTAN + HYDROCHLOROTHIAZIDE 5/160/25 | 18.41 | 31.13 | 8.79 | 58.33 | 41.86 | 2.54 | 1.82 | 16.47 | 28.24% | 0.72 | 28.2% |
| OLMESARTAN MEDOXOMIL + AMLODIPINE 40/5 | 27.93 | 18.41 |  | 46.34 | 25.81 | 2.90 | 1.61 | 20.53 | 44.30% | 1.28 | 44.3% |
| PERINDOPRIL + AMLODIPINE 10/10 | 21.75 | 18.41 |  | 40.16 | 24.91 | 2.51 | 1.56 | 15.25 | 37.97% | 0.95 | 38.0% |
| OLMESARTAN + AMLODIPINE + HYDROCHLOROTHIAZIDE 40/10/12.5 | 27.93 | 18.41 | 4.39 | 50.73 | 31.47 | 2.21 | 1.37 | 19.26 | 37.97% | 0.84 | 38.0% |
| PERINDOPRIL + AMLODIPINE 10/5 | 21.75 | 18.41 |  | 40.16 | 23.53 | 2.51 | 1.47 | 16.63 | 41.41% | 1.04 | 41.4% |
| OLMESARTAN + AMLODIPINE + HYDROCHLOROTHIAZIDE 40/5/12.5 | 27.93 | 18.41 | 4.39 | 50.73 | 30.65 | 2.21 | 1.33 | 20.08 | 39.58% | 0.87 | 39.6% |
| PERINDOPRIL + AMLODIPINE 5/10 | 19.79 | 18.41 |  | 38.20 | 21.87 | 2.39 | 1.37 | 16.33 | 42.75% | 1.02 | 42.7% |
| OLMESARTAN + AMLODIPINE + HYDROCHLOROTHIAZIDE 40/10/25 | 27.93 | 18.41 | 8.79 | 55.13 | 34.53 | 2.40 | 1.50 | 20.60 | 37.37% | 0.90 | 37.4% |
| OLMESARTAN + AMLODIPINE + HYDROCHLOROTHIAZIDE 40/5/25 | 27.93 | 18.41 | 8.79 | 55.13 | 33.77 | 2.40 | 1.47 | 21.36 | 38.74% | 0.93 | 38.7% |
| PERINDOPRIL + AMLODIPINE 5/5 | 19.79 | 18.41 |  | 38.20 | 20.51 | 2.39 | 1.28 | 17.69 | 46.31% | 1.11 | 46.3% |
| TELMISARTAN + AMLODIPINE 80/10 | 23.13 | 18.41 |  | 41.54 | 28.08 | 2.60 | 1.76 | 13.46 | 32.40% | 0.84 | 32.4% |
| RAMIPRIL + FELODIPINE 5/5 | 18.41 | 21.05 |  | 39.46 | 24.79 | 2.47 | 1.55 | 14.67 | 37.18% | 0.92 | 37.2% |
| TELMISARTAN + AMLODIPINE 80/5 | 23.13 | 18.41 |  | 41.54 | 26.71 | 2.60 | 1.67 | 14.83 | 35.70% | 0.93 | 35.7% |
| OLMESARTAN + AMLODIPINE 20/5 | 21.23 | 18.41 |  | 39.64 | 20.77 | 2.48 | 1.30 | 18.87 | 47.60% | 1.18 | 47.6% |
| ENALAPRIL + LERCANIDIPINE 10/10 | 20.23 | 19.73 |  | 39.96 | 24.59 | 2.50 | 1.54 | 15.37 | 38.46% | 0.96 | 38.5% |
| ENALAPRIL + LERCANIDIPINE 10/20 | 20.23 | 21.98 |  | 42.21 | 26.26 | 2.64 | 1.64 | 15.95 | 37.79% | 1.00 | 37.8% |
| RAMIPRIL + FELODIPINE 2.5/2.5 | 18.41 | 19.67 |  | 38.08 | 21.59 | 2.38 | 1.35 | 16.49 | 43.30% | 1.03 | 43.3% |
| OLMESARTAN + AMLODIPINE + HYDROCHLOROTHIAZIDE 20/5/12.5 | 21.23 | 18.41 | 4.39 | 44.03 | 37.96 | 1.91 | 1.65 | 6.07 | 13.79% | 0.26 | 13.8% |
| TELMISARTAN + AMLODIPINE 40/10 | 19.73 | 18.41 |  | 38.14 | 21.98 | 2.38 | 1.37 | 16.16 | 42.37% | 1.01 | 42.4% |
| TELMISARTAN + AMLODIPINE 40/5 | 19.73 | 18.41 |  | 38.14 | 20.64 | 2.38 | 1.29 | 17.50 | 45.88% | 1.09 | 45.9% |

**Table S1b. Absolute Cost Saving in AUD and percentage cost savings when single pill combinations are used instead of free-drug combinations, for patients and government, according to eligibility status of subsidy, for all 57 available single pill combinations**

|  | **Absolute Cost savings for patients (AUD)** | | | | **Absolute Cost Savings for Government (AUD)** | | | | **Percentage Cost savings for patients** | | | | **Percentage Cost savings for Government** | | | |
| --- | --- | --- | --- | --- | --- | --- | --- | --- | --- | --- | --- | --- | --- | --- | --- | --- |
| **Single Pill Combinations** | **General Before Safety Net** | **General After Safety Net** | **Con-cession Before Safety Net** | **Con-cession After Safety Net** | **General Before Safety Net** | **General After Safety Net** | **Con-cession Before Safety Net** | **Con-cession After Safety Net** | **General Before Safety Net** | **General After Safety Net** | **Con-cession Before Safety Net** | **Con-cession After Safety Net** | **General Before Safety Net** | **General After Safety Net** | **Con-cession Before Safety Net** | **Con-cession After Safety Net** |
| PERINDOPRIL + INDAPAMIDE 2.5/0.625 | 4.40 | 4.40 | 4.40 | 0.00 | 0.00 | 0.00 | 0.00 | 4.40 | 19.29% | 36.36% | 36.36% | 0.00% | 0.00% | 0.00% | 0.00% | 19.29% |
| ENALAPRIL + HYDRO-CHLOROTHIAZIDE 20/6 | -0.70 | 2.20 | 2.20 | 0.00 | 0.00 | - 2.90 | - 2.90 | - 0.70 | -3.04% | 22.22% | 22.22% | 0.00% | 0.00% | -22.09% | -22.09% | -3.04% |
| PERINDOPRIL + INDAPAMIDE 5/1.25 | 6.68 | 7.70 | 7.70 | 0.00 | 0.00 | - 1.02 | - 1.02 | 6.68 | 23.36% | 50.00% | 50.00% | 0.00% | 0.00% | -7.73% | -7.73% | 23.36% |
| PERINDOPRIL + INDAPAMIDE 4/1.25 | 6.68 | 7.70 | 7.70 | 0.00 | 0.00 | - 1.02 | - 1.02 | 6.68 | 23.36% | 50.00% | 50.00% | 0.00% | 0.00% | -7.73% | -7.73% | 23.36% |
| FOSINOPRIL + HYDRO-CHLOROTHIAZIDE 20/12.5 | 4.39 | 4.39 | 4.39 | 0.00 | -3.38 | - 3.38 | - 3.38 | 1.01 | 12.20% | 36.31% | 36.31% | 0.00% | -43.33% | -10.66% | -10.66% | 2.31% |
| VALSARTAN + HYDRO-CHLOROTHIAZIDE 80/12.5 | 3.51 | 4.39 | 4.39 | 0.00 | 0.00 | - 0.88 | - 0.88 | 3.51 | 11.07% | 36.31% | 36.31% | 0.00% | 0.00% | -4.48% | -4.48% | 11.07% |
| VALSARTAN + HYDRO-CHLOROTHIAZIDE 160/12.5 | 3.92 | 4.39 | 4.39 | 0.00 | 0.00 | - 0.47 | - 0.47 | 3.92 | 11.04% | 36.31% | 36.31% | 0.00% | 0.00% | -2.01% | -2.01% | 11.04% |
| VALSARTAN + HYDRO-CHLOROTHIAZIDE 320/12.5 | 4.39 | 4.39 | 4.39 | 0.00 | 0.06 | 0.06 | 0.06 | 4.45 | 12.20% | 36.31% | 36.31% | 0.00% | 1.37% | 0.21% | 0.21% | 11.02% |
| QUINAPRIL + HYDRO-CHLOROTHIAZIDE 20/12.5 | 4.39 | 4.39 | 4.39 | 0.00 | -3.00 | - 3.00 | - 3.00 | 1.39 | 12.20% | 36.31% | 36.31% | 0.00% | -68.81% | -10.62% | -10.62% | 3.44% |
| QUINAPRIL + HYDRO-CHLOROTHIAZIDE 10/12.5 | 4.39 | 4.39 | 4.39 | 0.00 | -2.92 | - 2.92 | - 2.92 | 1.47 | 12.20% | 36.31% | 36.31% | 0.00% | -111.45% | -11.01% | -11.01% | 3.81% |
| OLMESARTAN + HYDRO-CHLOROTHIAZIDE 40/12.5 | 4.05 | 4.39 | 4.39 | 0.00 | 0.00 | - 0.34 | - 0.34 | 4.05 | 12.53% | 36.31% | 36.31% | 0.00% | 0.00% | -1.68% | -1.68% | 12.53% |
| OLMESARTAN + HYDRO-  CHLOROTHIAZIDE 20/12.5 | 4.03 | 4.39 | 4.39 | 0.00 | 0.00 | - 0.36 | - 0.36 | 4.03 | 15.73% | 36.31% | 36.31% | 0.00% | 0.00% | -2.66% | -2.66% | 15.73% |
| TELMISARTAN + HYDRO-CHLOROTHIAZIDE 80/12.5 | 4.39 | 4.39 | 4.39 | 0.00 | 0.00 | 0.00 | 0.00 | 4.39 | 15.95% | 36.31% | 36.31% | 0.00% | 0.00% | 0.00% | 0.00% | 15.95% |
| VALSARTAN + HYDRO-CHLOROTHIAZIDE 320/25 | 8.79 | 7.70 | 7.70 | 0.00 | -2.27 | - 1.18 | - 1.18 | 6.52 | 21.76% | 50.00% | 50.00% | 0.00% | -51.71% | -4.02% | -4.02% | 14.56% |
| VALSARTAN + HYDRO-CHLOROTHIAZIDE 160/25 | 8.32 | 7.70 | 7.70 | 0.00 | -2.35 | - 1.73 | - 1.73 | 5.97 | 20.84% | 50.00% | 50.00% | 0.00% | 0.00% | -7.06% | -7.06% | 14.95% |
| CANDESARTAN + HYDRO-CHLOROTHIAZIDE 32/12.5 | 4.39 | 4.39 | 4.39 | 0.00 | 0.00 | 0.00 | 0.00 | 4.39 | 16.90% | 36.31% | 36.31% | 0.00% | 0.00% | 0.00% | 0.00% | 16.90% |
| EPROSARTAN + HYDRO-CHLOROTHIAZIDE 600/12.5 | 4.39 | 4.39 | 4.39 | 0.00 | -10.72 | - 10.72 | - 10.72 | - 6.33 | 12.20% | 36.31% | 36.31% | 0.00% | -82.97% | -29.11% | -29.11% | -12.94% |
| CANDESARTAN + HYDRO-CHLOROTHIAZIDE 16/12.5 | 4.05 | 4.39 | 4.39 | 0.00 | 0.00 | - 0.34 | - 0.34 | 4.05 | 15.80% | 36.31% | 36.31% | 0.00% | 0.00% | -2.51% | -2.51% | 15.80% |
| TELMISARTAN + HYDRO-CHLOROTHIAZIDE 40/12.5 | 4.14 | 4.39 | 4.39 | 0.00 | 0.00 | - 0.25 | - 0.25 | 4.14 | 17.16% | 36.31% | 36.31% | 0.00% | 0.00% | -2.08% | -2.08% | 17.16% |
| IRBESARTAN + HYDRO-CHLOROTHIAZIDE 300/12.5 | 4.17 | 4.39 | 4.39 | 0.00 | 0.00 | - 0.22 | - 0.22 | 4.17 | 16.75% | 36.31% | 36.31% | 0.00% | 0.00% | -1.72% | -1.72% | 16.75% |
| IRBESARTAN + HYDRO-CHLOROTHIAZIDE 150/12.5 | 4.39 | 4.39 | 4.39 | 0.00 | 0.00 | 0.00 | 0.00 | 4.39 | 19.25% | 36.31% | 36.31% | 0.00% | 0.00% | 0.00% | 0.00% | 19.25% |
| OLMESARTAN + HYDRO-CHLOROTHIAZIDE 40/25 | 6.45 | 7.70 | 7.70 | 0.00 | 0.00 | - 1.25 | - 1.25 | 6.45 | 17.57% | 50.00% | 50.00% | 0.00% | 0.00% | -5.86% | -5.86% | 17.57% |
| TELMISARTAN + HYDRO-CHLOROTHIAZIDE 80/25 | 8.08 | 7.70 | 7.70 | 0.00 | 0.00 | 0.38 | 0.38 | 8.08 | 25.31% | 50.00% | 50.00% | 0.00% | 0.00% | 2.30% | 2.30% | 25.31% |
| CANDESARTAN + HYDRO-CHLOROTHIAZIDE 32/25 | 7.71 | 7.70 | 7.70 | 0.00 | 0.00 | 0.01 | 0.01 | 7.71 | 25.38% | 50.00% | 50.00% | 0.00% | 0.00% | 0.07% | 0.07% | 25.38% |
| IRBESARTAN + HYDRO-CHLOROTHIAZIDE 300/25 | 7.89 | 7.70 | 7.70 | 0.00 | 0.00 | 0.19 | 0.19 | 7.89 | 26.93% | 50.00% | 50.00% | 0.00% | 0.00% | 1.37% | 1.37% | 26.93% |
| AMLODIPINE + VALSARTAN 10/320 | 18.41 | 7.70 | 7.70 | 0.00 | -5.38 | 5.33 | 5.33 | 13.03 | 36.81% | 50.00% | 50.00% | 0.00% | -122.55% | 13.67% | 13.67% | 23.95% |
| AMLODIPINE + VALSARTAN 5/320 | 18.41 | 7.70 | 7.70 | 0.00 | -4.93 | 5.78 | 5.78 | 13.48 | 36.81% | 50.00% | 50.00% | 0.00% | -112.30% | 14.82% | 14.82% | 24.78% |
| TRANDOLAPRIL + VERAPAMIL 4/240 | 31.60 | 7.70 | 7.70 | 0.00 | -7.43 | 16.47 | 16.47 | 24.17 | 50.00% | 50.00% | 50.00% | 0.00% | -72.70% | 28.39% | 28.39% | 32.92% |
| AMLODIPINE + VALSARTAN 10/160 | 17.94 | 7.70 | 7.70 | 0.00 | -4.90 | 5.34 | 5.34 | 13.04 | 36.21% | 50.00% | 50.00% | 0.00% | 0.00% | 15.64% | 15.64% | 26.32% |
| AMLODIPINE + VALSARTAN 5/160 | 17.94 | 7.70 | 7.70 | 0.00 | -3.77 | 6.47 | 6.47 | 14.17 | 36.21% | 50.00% | 50.00% | 0.00% | 0.00% | 18.95% | 18.95% | 28.60% |
| AMLODIPINE + VALSARTAN + HYDROCHLOROTHIAZIDE 10/320/25 | 27.20 | 15.40 | 15.40 | 0.00 | -11.87 | - 0.07 | - 0.07 | 15.33 | 46.26% | 66.67% | 66.67% | 0.00% | -270.39% | -0.17% | -0.17% | 24.26% |
| AMLODIPINE + VALSARTAN + HYDROCHLOROTHIAZIDE 10/160/12.5 | 22.33 | 12.09 | 12.09 | 0.00 | -8.14 | 2.10 | 2.10 | 14.19 | 41.41% | 61.09% | 61.09% | 0.00% | 0.00% | 6.15% | 6.15% | 26.31% |
| AMLODIPINE + VALSARTAN + HYDROCHLOROTHIAZIDE 5/160/12.5 | 22.33 | 12.09 | 12.09 | 0.00 | -7.03 | 3.21 | 3.21 | 15.30 | 41.41% | 61.09% | 61.09% | 0.00% | 0.00% | 9.40% | 9.40% | 28.37% |
| AMLODIPINE + VALSARTAN 5/80 | 14.72 | 7.70 | 7.70 | 0.00 | 0.00 | 7.02 | 7.02 | 14.72 | 32.18% | 50.00% | 50.00% | 0.00% | 0.00% | 23.14% | 23.14% | 32.18% |
| AMLODIPINE + VALSARTAN + HYDROCHLOROTHIAZIDE 10/160/25 | 26.73 | 15.40 | 15.40 | 0.00 | -11.40 | - 0.07 | - 0.07 | 15.33 | 45.83% | 66.67% | 66.67% | 0.00% | 0.00% | -0.20% | -0.20% | 26.28% |
| OLMESARTAN + AMLODIPINE 40/10 | 19.61 | 7.70 | 7.70 | 0.00 | 0.00 | 11.91 | 11.91 | 19.61 | 42.32% | 50.00% | 50.00% | 0.00% | 0.00% | 38.49% | 38.49% | 42.32% |
| TRANDOLAPRIL + VERAPAMIL 2/180 | 16.23 | 7.70 | 7.70 | 0.00 | -4.65 | 3.88 | 3.88 | 11.58 | 33.93% | 50.00% | 50.00% | 0.00% | 0.00% | 11.96% | 11.96% | 24.21% |
| AMLODIPINE + VALSARTAN + HYDROCHLOROTHIAZIDE 5/160/25 | 26.73 | 15.40 | 15.40 | 0.00 | -10.26 | 1.07 | 1.07 | 16.47 | 45.83% | 66.67% | 66.67% | 0.00% | 0.00% | 3.04% | 3.04% | 28.24% |
| OLMESARTAN MEDOXOMIL + AMLODIPINE 40/5 | 20.53 | 7.70 | 7.70 | 0.00 | 0.00 | 12.83 | 12.83 | 20.53 | 44.30% | 50.00% | 50.00% | 0.00% | 0.00% | 41.47% | 41.47% | 44.30% |
| PERINDOPRIL + AMLODIPINE 10/10 | 15.25 | 7.70 | 7.70 | 0.00 | 0.00 | 7.55 | 7.55 | 15.25 | 37.97% | 50.00% | 50.00% | 0.00% | 0.00% | 30.49% | 30.49% | 37.97% |
| OLMESARTAN + AMLODIPINE + HYDROCHLOROTHIAZIDE 40/10/12.5 | 19.26 | 15.40 | 15.40 | 0.00 | 0.00 | 3.86 | 3.86 | 19.26 | 37.97% | 66.67% | 66.67% | 0.00% | 0.00% | 13.97% | 13.97% | 37.97% |
| PERINDOPRIL + AMLODIPINE 10/5 | 16.63 | 7.70 | 7.70 | 0.00 | 0.00 | 8.93 | 8.93 | 16.63 | 41.41% | 50.00% | 50.00% | 0.00% | 0.00% | 36.07% | 36.07% | 41.41% |
| OLMESARTAN + AMLODIPINE + HYDROCHLOROTHIAZIDE 40/5/12.5 | 20.08 | 12.09 | 12.09 | 0.00 | 0.00 | 7.99 | 7.99 | 20.08 | 39.58% | 61.09% | 61.09% | 0.00% | 0.00% | 25.82% | 25.82% | 39.58% |
| PERINDOPRIL + AMLODIPINE 5/10 | 16.33 | 7.70 | 7.70 | 0.00 | 0.00 | 8.63 | 8.63 | 16.33 | 42.75% | 50.00% | 50.00% | 0.00% | 0.00% | 37.85% | 37.85% | 42.75% |
| OLMESARTAN + AMLODIPINE + HYDROCHLOROTHIAZIDE 40/10/25 | 23.53 | 15.40 | 15.40 | 0.00 | -2.93 | 5.20 | 5.20 | 20.60 | 42.68% | 66.67% | 66.67% | 0.00% | 0.00% | 16.23% | 16.23% | 37.37% |
| OLMESARTAN + AMLODIPINE + HYDROCHLOROTHIAZIDE 40/5/25 | 23.53 | 15.40 | 15.40 | 0.00 | -2.17 | 5.96 | 5.96 | 21.36 | 42.68% | 66.67% | 66.67% | 0.00% | 0.00% | 18.61% | 18.61% | 38.74% |
| PERINDOPRIL + AMLODIPINE 5/5 | 17.69 | 7.70 | 7.70 | 0.00 | 0.00 | 9.99 | 9.99 | 17.69 | 46.31% | 50.00% | 50.00% | 0.00% | 0.00% | 43.82% | 43.82% | 46.31% |
| TELMISARTAN + AMLODIPINE 80/10 | 13.46 | 7.70 | 7.70 | 0.00 | 0.00 | 5.76 | 5.76 | 13.46 | 32.40% | 50.00% | 50.00% | 0.00% | 0.00% | 22.04% | 22.04% | 32.40% |
| RAMIPRIL + FELODIPINE 5/5 | 14.67 | 7.70 | 7.70 | 0.00 | 0.00 | 6.97 | 6.97 | 14.67 | 37.18% | 50.00% | 50.00% | 0.00% | 0.00% | 28.97% | 28.97% | 37.18% |
| TELMISARTAN + AMLODIPINE 80/5 | 14.83 | 7.70 | 7.70 | 0.00 | 0.00 | 7.13 | 7.13 | 14.83 | 35.70% | 50.00% | 50.00% | 0.00% | 0.00% | 27.28% | 27.28% | 35.70% |
| OLMESARTAN + AMLODIPINE 20/5 | 18.87 | 7.70 | 7.70 | 0.00 | 0.00 | 11.17 | 11.17 | 18.87 | 47.60% | 50.00% | 50.00% | 0.00% | 0.00% | 46.08% | 46.08% | 47.60% |
| ENALAPRIL + LERCANIDIPINE 10/10 | 15.37 | 7.70 | 7.70 | 0.00 | 0.00 | 7.67 | 7.67 | 15.37 | 38.46% | 50.00% | 50.00% | 0.00% | 0.00% | 31.23% | 31.23% | 38.46% |
| ENALAPRIL + LERCANIDIPINE 10/20 | 15.95 | 7.70 | 7.70 | 0.00 | 0.00 | 8.25 | 8.25 | 15.95 | 37.79% | 50.00% | 50.00% | 0.00% | 0.00% | 30.77% | 30.77% | 37.79% |
| RAMIPRIL + FELODIPINE 2.5/2.5 | 16.49 | 7.70 | 7.70 | 0.00 | 0.00 | 8.79 | 8.79 | 16.49 | 43.30% | 50.00% | 50.00% | 0.00% | 0.00% | 38.76% | 38.76% | 43.30% |
| OLMESARTAN + AMLODIPINE + HYDROCHLOROTHIAZIDE 20/5/12.5 | 12.43 | 12.09 | 12.09 | 0.00 | -6.36 | - 6.02 | - 6.02 | 6.07 | 28.23% | 61.09% | 61.09% | 0.00% | 0.00% | -24.83% | -24.83% | 13.79% |
| TELMISARTAN + AMLODIPINE 40/10 | 16.16 | 7.70 | 7.70 | 0.00 | 0.00 | 8.46 | 8.46 | 16.16 | 42.37% | 50.00% | 50.00% | 0.00% | 0.00% | 37.20% | 37.20% | 42.37% |
| TELMISARTAN + AMLODIPINE 40/5 | 17.50 | 7.70 | 7.70 | 0.00 | 0.00 | 9.80 | 9.80 | 17.50 | 45.88% | 50.00% | 50.00% | 0.00% | 0.00% | 43.10% | 43.10% | 45.88% |

**Figure S1. Price reduction in AUD for supply of 60 days of medicines using single pill combinations instead of free-drug combinations, for 57 available single pill combinations**

**Figure S2. Price reduction per mmHg blood pressure lowering when using single pill combinations instead of free-drug combinations for the 57 different combinations**

**CHEERS 2022 Checklist**

**First-line treatment of hypertension with single pill combinations: a simple strategy to save costs for the patient and government**

|  | **Item** | **Guidance for Reporting** | **Reported in section** |
| --- | --- | --- | --- |
| **TITLE** | | |  |
| Title | 1 | Identify the study as an economic evaluation and specify the interventions being compared. | Page 1 |
| **ABSTRACT** | | |  |
| Abstract | 2 | Provide a structured summary that highlights context, key methods, results and alternative analyses. | Page 2 |
| **INTRODUCTION** | | |  |
| Background and objectives | 3 | Give the context for the study, the study question and its practical relevance for decision making in policy or practice. | Page 3-4 |
| **METHODS** | | |  |
| Health economic  analysis plan | 4 | Indicate whether a health economic analysis plan was developed and  where available. | Not applicable |
| Study population | 5 | Describe characteristics of the study population (such as age range, demographics, socioeconomic, or clinical characteristics). | Page 5 |
| Setting and location | 6 | Provide relevant contextual information that may influence findings. | Page 5 |
| Comparators | 7 | Describe the interventions or strategies being compared and why chosen. | Page 5 |
| Perspective | 8 | State the perspective(s) adopted by the study and why chosen. | Not applicable |
| Time horizon | 9 | State the time horizon for the study and why appropriate. | Not applicable |
| Discount rate | 10 | Report the discount rate(s) and reason chosen. | Not applicable |
| Selection of outcomes | 11 | Describe what outcomes were used as the measure(s) of benefit(s) and harm(s). | Page 5-6 |
| Measurement of outcomes | 12 | Describe how outcomes used to capture benefit(s) and harm(s) were measured. | Page 5-6 |
| Valuation of outcomes | 13 | Describe the population and methods used to measure and value outcomes. | Page 5-6 |
| Measurement and valuation of resources  and costs | 14 | Describe how costs were valued. | Page 5-6 |
| Currency, price date, and conversion | 15 | Report the dates of the estimated resource quantities and unit costs, plus the currency and year of conversion. | Page 5 |
| Rationale and  description of model | 16 | If modelling is used, describe in detail and why used. Report if the model  is publicly available and where it can be accessed. | Not applicable |
| Analytics and assumptions | 17 | Describe any methods for analysing or statistically transforming data, any extrapolation methods, and approaches for validating any model used. | Page 5-6 |
| Characterizing heterogeneity | 18 | Describe any methods used for estimating how the results of the study vary for sub-groups. | Page 5-6 |
| Characterizing  distributional effects | 19 | Describe how impacts are distributed across different individuals  or adjustments made to reflect priority populations. | Not applicable |
| Characterizing uncertainty | 20 | Describe methods to characterize any sources of uncertainty in the analysis. | Not applicable |
| Approach to engagement with patients and others affected by the study | 21 | Describe any approaches to engage patients or service recipients, the general public, communities, or stakeholders (e.g., clinicians or payers) in the design of the study. | Not applicable |
| **RESULTS** | | |  |
| Study parameters | 22 | Report all analytic inputs (e.g., values, ranges, references) including uncertainty or distributional assumptions. | Page 6-8 |
| Summary of main results | 23 | Report the mean values for the main categories of costs and outcomes of interest and summarise them in the most appropriate overall measure. | Page 6-8 |
| Effect of uncertainty | 24 | Describe how uncertainty about analytic judgments, inputs, or projections  affect findings. Report the effect of choice of discount rate and time horizon, if applicable. | Not applicable |
| Effect of engagement with patients and others affected by the study | 25 | Report on any difference patient/service recipient, general public, community, or stakeholder involvement made to the approach or findings of the study | Not applicable |
| **DISCUSSION** | | |  |
| Study findings, limitations, generalizability, and current knowledge | 26 | Report key findings, limitations, ethical or equity considerations not captured, and how these could impact patients, policy, or practice. | Page 8-12 |
| **OTHER RELEVANT INFORMATION** | | | |
| Source of funding | 27 | Describe how the study was funded and any role of the funder in the identification, design, conduct, and reporting of the analysis | Page 1 |
| Conflicts of interest | 28 | Report authors conflicts of interest according to journal or  International Committee of Medical Journal Editors requirements. | Page 1 |

Husereau D, Drummond M, Augustovski F, de Bekker-Grob E, Briggs AH, Carswell C, Caulley L, Chaiyakunapruk N, Greenberg D, Loder E, Mauskopf J, Mullins CD, Petrou S, Pwu RF, Staniszewska S; CHEERS 2022 ISPOR Good Research Practices Task Force. Consolidated Health Economic Evaluation Reporting Standards 2022 (CHEERS 2022) Statement: Updated Reporting Guidance for Health Economic Evaluations. BMJ. 2022;376:e067975.

The checklist is Open Access distributed in accordance with the terms of the Creative Commons Attribution (CC BY 4.0) license, which permits others to distribute, remix, adapt and build upon this work, for commercial use, provided the original work is properly cited. See: [http://creativecommons.org/licenses/by/4.0/.](http://creativecommons.org/licenses/by/4.0/)
